# Retraction Note: Optimization of oncolytic effect of Newcastle disease virus Clone30 by selecting sensitive tumor host and constructing more oncolytic viruses

**DOI:** 10.1038/s41434-024-00459-9

**Published:** 2024-07-04

**Authors:** Tianyan Liu, Yu Zhang, Yukai Cao, Shan Jiang, Rui Sun, Jiechao Yin, Zhenqiu Gao, Guiping Ren, Zhenzhong Wang, Qingzhong Yu, Guangchao Sui, Xu Sun, Wenying Sun, Wei Xiao, Deshan Li

**Affiliations:** 1 College of Life Science, Northeast Agricultural University, Harbin, China; 2 Jiangsu Kanion Pharmaceutical Co. LTD, State Key Laboratory of New-tech for Chinese Medicine Pharmaceutical Process, Lianyungang, Jiangsu China; 3 School of Pharmacy, Yancheng Teachers University, Yancheng, China; 4 Department of Agriculture, Southeast Poultry Research Laboratory, Agricultural Research Service, Athens, GA USA; 5 College of Life Science, Northeast Forestry University, Harbin, China; 6 Present Address: College of Life Science, Northeast Agricultural University, Harbin, China

Retraction Note to: *Gene Therapy* 10.1038/s41434-020-0145-9, published online 14 May 2020

The Editor-in-Chief has retracted this article. After publication, concerns were raised regarding similarities in some of the images presented in the figures, specifically:

- In Fig. 2A, the SH-SY5Y rClone30-RFP and rAnh-RFP images appear to share a highly similar feature.
- In Fig. 4A, DAPI HepG2 panels rClone30 and rClone30-Anh(HN), Normal MDA-MB-231 panels rClone30 and rClone30-Anh(HN), and Normal A549 panels rClone30-Anh(F) and rAnh appear to overlap.
- In Fig. 6A, some of the tumours exhibit similar features.
- In Supplementary Fig. 2B, H22 DAPI panels rClone30-Anh(F) and rClone30-Anh(HN-F) appear to overlap.

The authors have stated that the original data are no longer available. Due to the high number of image concerns, the Editor-in-Chief no longer has confidence in the presented data.

Tianyan Liu, Qingzhong Yu and Deshan Li do not agree to this retraction. Guangchao Sui agrees to this retraction. Yu Zhang, Yukai Cao, Shan Jiang, Rui Sun, Jiechao Yin, Zhenqiu Gao, Guiping Ren, Zhenzhong Wang, Xu Sun, Wenying Sun and Wei Xiao have not responded to any correspondence from the editor or publisher about this retraction.

